# AppraisalCloudPCT: A Computational Model of Emotions for Socially Interactive Robots for Autistic Rehabilitation

**DOI:** 10.1155/2023/5960764

**Published:** 2023-03-07

**Authors:** Ting Yan, Shengzhao Lin, Jinfeng Wang, Fuhao Deng, Zijian Jiang, Gong Chen, Jionglong Su, Jiaming Zhang

**Affiliations:** ^1^The Brain Cognition and Brain Disease Institute, Shenzhen Institute of Advanced Technology, Chinese Academy of Sciences, Shenzhen, Guangdong 518055, China; ^2^Institute of Robotics and Intelligent Manufacturing, the Chinese University of Hong Kong (Shenzhen), Shenzhen, Guangdong 518172, China; ^3^Department of Mathematical Sciences, Xi'an Jiaotong-Liverpool University, Suzhou, Jiangsu 215123, China; ^4^Shenzhen TOP Intelligent Manufacturing and Technology Co., Ltd., Shenzhen, Guangdong 518129, China; ^5^Sunwoda Electronic Co., Ltd., Shiyan Street, Bao'an District, Shenzhen 518000, China; ^6^School of AI and Advanced Computing, XJTLU Entrepreneur College (Taicang), Xi'an Jiaotong-Liverpool University, Suzhou, Jiangsu 215123, China

## Abstract

Computational models of emotions can not only improve the effectiveness and efficiency of human-robot interaction but also coordinate a robot to adapt to its environment better. When designing computational models of emotions for socially interactive robots, especially for robots for people with special needs such as autistic children, one should take into account the social and communicative characteristics of such groups of people. This article presents a novel computational model of emotions called AppraisalCloudPCT that is suitable for socially interactive robots that can be adopted in autistic rehabilitation which, to the best of our knowledge, is the first computational model of emotions built for robots that can satisfy the needs of a special group of people such as autistic children. To begin with, some fundamental and notable computational models of emotions (e.g., OCC, Scherer's appraisal theory, PAD) that have deep and profound influence on building some significant models (e.g., PRESENCE, iGrace, xEmotion) for socially interactive robots are revisited. Then, a comparative assessment between our AppraisalCloudPCT and other five significant models for socially interactive robots is conducted. Great efforts have been made in building our proposed model to meet all of the six criteria for comparison, by adopting the appraisal theories on emotions, perceptual control theory on emotions, a component model view of appraisal models, and cloud robotics. Details of how to implement our model in a socially interactive robot we developed for autistic rehabilitation are also elaborated in this article. Future studies should examine how our model performs in different robots and also in more interactive scenarios.

## 1. Introduction

Before probing into the scope of computational models of emotions, it is necessary to understand the emotion terminology for the purpose of clarity. To begin with, six terms (i.e., affect, appraisal, cognition, emotion, feeling, and mood) are defined in [1] as follows: (1) to inform one or more cognitive processes, affect which is any information (feeling, mood, and emotion) is used; (2) the process of making judgments (appraisals) about the relationship between an individual's beliefs, desires, and intentions [2] and perceived events is defined as appraisal; (3) mental processes associated with the comprehension, acquisition, and alteration of knowledge are defined as cognition, such as planning, learning, inference, and recall; (4) owing to concepts and states, emotion is used to inform responses and is defined as cognitive data generated by events (internal and external); (5) the subjective experience of an emotion or of a series of emotions is defined as feeling; (6) mood is the general state of an emotion that lasts longer and is less variable than the emotion itself.

As for the terminology computational models, according to Simon [3], by drawing results from a system's premises (for example, a weather forecast system), and by predicting the system's behavior, computational models can simulate a system. Furthermore, it was argued that given some premises and appraisal operations, computational models of emotion can predict and potentially produce behavior [4]. According to Marsella et al. [5], computational models of emotions play various roles in research and applications: (1) from the perspective of psychological research, computational models better support our understanding of human emotional processes; (2) from the perspective of AI and robotics research, modeling of emotion can influence the reasoning process or coordinate an agent or robot to better adapt to its environment; (3) from the perspective of HCI research, modeling of emotion improves the efficiency and effectiveness in interaction, as well as enhancing user experience.

To investigate the importance of affective processes in social development and socially situated learning of robots coexisting with humans in the human environment, the computational models of emotions for socially interactive robots were introduced [6]. According to Breazeal et al. [7], to effectively engage in emotion-based interactions, robots must possess three kinds of capabilities: (1) to recognize and interpret human emotional signals, (2) to operate by means of their internal emotional models that are often based on theories in psychology, and (3) to communicate their affective states to others. Since the emotional responses of a robot can be determined by the robot's computational model of emotion which depends on the interaction of its internal cognitive-affective state with the external environment [7], these internal emotional models are crucial for human-robot interactions [8].

Many people with autism spectrum disorder (ASD) have characteristics such as difficulty in social communication (e.g., poor perception of nonverbal cues including facial expressions and gestures in body language, as well as inappropriate expressions), limited and repeated behaviors, as well as narrow, focused interests (in Diagnostic and Statistical Manual of Mental Disorders 5th Edition: DSM 5 [9]). It is increasingly necessary to introduce social interactive robots as an auxiliary means for the treatment and rehabilitation of ASD, so as to improve the diversity of treatment and the effectiveness of rehabilitation training, and to mitigate the medical staff shortages in mainland China and the rest of the world [10]. A number of treatment and training targets, such as triadic interactions, joint attention (JA), turn-taking activities, improving eye contact and self-initiated interactions, assisting the diagnostic process, emotion recognition, and imitation, can be achieved by robotics for autism [11]. Moreover, robots have demonstrated their potentials in 24 of 74 ASD objectives in eight domains, including preschool skills for children with ASD, motor experiences and skills, social/interpersonal interactions and relations, emotional wellbeing, functioning in daily reality, sensory experiences, and coping, play, and communication [12].

However, better utilization of robots and HCI for autism intervention in the clinical setting does not necessarily lead to robots that are clinically more useful for ASD intervention [13]. This is partly due to the difficulty in the ASD patients to understand the emotional and mental states of others, a feature of the autism spectrum conditions (ASC) [14]. ASC patients show symptoms of stunted development in their ability to recognize and differentiate between different emotional expressions [15]. In addition, children with ASD may be focused on objects of interest for a very long period of time, failing to deliver rehabilitation training outcomes. Hence, if robots are able to follow the gaze, they may be deployed for human-robot interaction tasks, including rehabilitation training for autism [16, 17].

Consequently, when designing computational models of emotions for socially interactive robots, especially for robots for a special group of people such as autistic children, one should take into account the social and communicative characteristics of such a group of people. There are four world-leading research groups with pioneering work in promoting social robots as useful tools in autism therapy, including the Kerstin Dautenhahn Group [18–20]), the Ayanna Howard Group [21, 22], the Maja Matarić Group [23–25], and the Bram Vanderborght Group [26, 27]. However, none of the 4 research groups have designed or applied computational models of emotions for the social robots used in their autism therapy studies. Therefore, this article will propose a novel computational model of emotions that are suitable for and can implement in socially interactive robots, especially for robots adopted in autistic rehabilitation.

The contributions of this article are threefold. First and most importantly, this article presents a novel computational model of emotions so-called AppraisalCloudPCT that is suitable for socially interactive robots that may be used in autistic rehabilitation which, to the best of our knowledge, is the first computational model of emotions built for robots that can satisfy the needs of a special group of people such as autistic children. Second, such a computational model of emotions can enhance human-robot interaction more interactively and effectively, as it takes into account a user's intention and attention and can coordinate the robot to make an appropriate response to the surrounding emotional environment. Third, such a computational model of emotions can achieve a high degree of simulation of human emotions and can be computationally implementable in various robots, as our proposed computational model of emotion is based on the appraisal theories on emotions [28–31], perceptual control theory on emotions [32], a compositional view of model building [5], and cloud robotics [33, 34]/cloud medical robots [35–38].

The rest of the paper is organized as follows.  Section 2 revisits some fundamental and notable computational models of emotions that have a deep and profound influence on building some significant computational models of emotions for socially interactive robots, which will be reviewed in Section 3.  Section 4 presents our proposed computational model of robotic emotions so-called AppraisalCloudPCT, and its implementation in a social robot for autistic rehabilitation will be elaborated in Section 5. Finally, the conclusions, limitations, discussion, and future work are given in Section 6.

## 2. Classical Computational Models of Emotions

The development of computational modeling of emotion and cognition has been accelerated by recent human cognitive and psychological studies related to emotion [1]. For example, according to Marsella et al. [5], concepts drawn from AI have been cast in the appraisal theory of several computational models, including the belief-desire-intention (BDI) model, fuzzy logic, knowledge representation, Q-learning, planning, neural networks, and decision-making.

Marsella et al. [5] used a figure of a “family tree” of a number of the theoretical traditions and significant models (e.g., rational theories, anatomical, dimensional, and appraisal) to illustrate from which they stem. Instead of using a “family tree,” Lin et al. [1] used two tables to review the fundamental theoretical traditions of emotion and cognition and effects modeled by some well-known computational models.

As this article will not focus on the interaction between emotion and cognition as [1] did, cognition theories such as the BDI model will not be reviewed here. Rather, appraisal theories such as OCC (the affect-derivation model proposed by Ortony et al. [39]) and Scherer's appraisal theory [40], as well as dimensional theories of emotion such as PAD [41], will be revisited here as classical theoretical traditions listed in [5]. Other theories, such as perceptual control theory on emotions [32] and a compositional view of model building [5], which were not listed in the “family tree” in [5], will be also revisited.

### 2.1. OCC

Ortony et al. [39] proposed an appraisal theory, i.e., the OCC theory in their book “The Cognitive Structure of Emotions,” in which 22 emotions are categorized based on the appraisal of intensity (arousal) and pleasure/displeasure (valence). The OCC theory offers a structure for variables such as the familiarity of an object or the likelihood of an event, to determine the intensity of the emotion types. Based on what is being appraised, the OCC theory broke down the valence appraisal into three categories: praiseworthiness (of an action), like/dislike (of an entity), and desirability (of an event). In addition, when some branches are combined, well-being/attribution compound emotions (e.g., remorse and gratitude) concerning the consequences of events caused by an agent's actions will be formed.

Specifications have three elements (i.e., the type specification stating the conditions that trigger an emotion of the type, a list of tokens, and a list of variables affecting intensity for each emotion type) that are given for each of the 22 emotion types. The list of tokens specifies which emotional words can be classified as belonging to the type of emotion discussed.

Five negative categories (hate, fear, distress, anger, and disappointment remorse) and five positive categories (love, relief, hope, joy, gratitude, and pride) from the OCC model were proposed to use in Ortony [42], in order to decrease the complexity for the development of believable characters. However, for a character using facial expressions only, such ten emotional categories might still be too much, as argued by Bartneck [43], and he proposed to split the emotion process of the OCC model into five phases. Additionally, to resolve the ambiguities identified in the OCC model, a new view of the emotional logic structure of the OCC model based on inheritance was proposed by Steunebrink et al. [44].

### 2.2. Scherer's Appraisal Theory

Appraisal theories of emotion, first introduced by Arnold [45] and Lazarus [2, 46], are rooted in Aristotle, Descartes, Spinoza, and Hume [47]. Ellsworth and Scherer and their students actively developed them [28, 40, 48–50] in the early 1980s (see the historical reviews by Scherer [40, 51]). Appraisal theories of emotion relate emotions to the more immediate cognitive assessment of coping capabilities, causal attribution, and evaluation of meaning [52], while the evolutionary theories of emotion relate emotions to biological adaption in the distant past by contrast. Clore and Ortony [53] treated appraisals to be the psychological representations of emotional significance for the person experiencing the emotion. And Scherer [51] reviewed a central tenet of appraisal theory and arrived at the conclusion that through some dimensions or criteria emotions are triggered and distinguished based on one's subjective evaluation of personal significance in events, objects, or situations.

Scherer [31] used stimulus evaluation checks (SECs) and defined in the component process model of emotion (CPM) [40, 48, 54–56], to represent the minimum dimension or criteria set sufficient and necessary in distinguishing the essential families of emotional states. The changes in the states of most if not all of the five organismic subsystems will respond to the assessments of external or internal stimuli related to the organism's primary concerns, and such an episode of interrelated, synchronized changes is defined as emotion [40] in the framework of the CPM (see Figure 1 in [50]). According to CPM, emotion is considered to be a theoretical structure, consisting of five components, each corresponding to one of the five unique functions [50]. In the light of CPM, SECs are processed in sequence of a fixed order, containing four stages in the appraisal process each corresponding to one of the four appraisal objectives, i.e., relevance, implications, coping potential, and normative significance [47]. Moreover, CPM assumes that changes in the internal or external events keep maintaining a recursive appraisal process until the monitoring subsystem sends a signal to terminate or adjust the stimulation triggering the appraisal episode initially [40, 50].

In summary, appraisal theories of emotion not only can be used to investigate the origin of emotion but can also be used to account for the emotions of people experiencing feelings, using the Geneva Emotion Wheel (see the second version in [57]), or the Geneva Expert System on Emotion (https://www.unige.ch/cisa/properemo/gep17/intro1.php). In addition, facial expressions and physiological processes may change during the evaluation or appraisal of the personal significance of a certain object or situation, but which discrete emotion is experienced can be determined by the specific profile of appraisal (i.e., the antecedent of the emotion), according to Niedenthal et al. [52]. As a result, two individuals can experience different emotions despite being subjected to the same event or stimulus, which is consistent with the appraisal theories of emotion.

### 2.3. PAD

According to dimensional theories of emotion, emotion and other affective phenomena should be classified and labeled in the way of the social construction–as points in continuous (usually two- or three-dimensional) space but not as discrete entities [41, 58–60]. The historical development of dimensional theories of emotion can be traced back to James [61], Schachter and Singer [62], Russell [58], and Barrett [59]. Russell [63] suggested replacing discrete emotions with core affect due to cross-cultural differences which attribute specific emotions to facial expressions. Scarantino [64] described the “core affect” as follows:

“Core affect, understood as the category comprising the set of all possible valence and arousal combinations on the circumplex, differs from discrete emotions in three crucial ways: it is ubiquitous, it is objectless, and it is primitive.” ([64], p. 948).

According to Russell ([58], p. 154), a person is in exactly one affective state at any time and such possible core affective states can be characterized in the space of continuous and broad dimensions. Mehrabian and Russell's “PAD” model [41] consists of three dimensions corresponding to pleasure (measuring valence), arousal (to measure the level of affective activation), and dominance (a measure of control or power), respectively. Many computational models of emotion were inspired by the PAD model, such as WASABI [65], a PAD-based model of core affects incorporating Scherer's sequential-checking theory.

### 2.4. Perceptual Control Theory on Emotions

Perceptual control theory (PCT) [66] is a theory on how living organisms can control their inputs instead of their outputs. The idea of PCT can be attributed to [67]: “What we have is a circuit, not an arc or broken segment of a circle. This circuit is more truly termed organic than reflex because the motor response determines the stimulus, just as truly as sensory stimulus determines the movement ([67]; p. 363).” “PCT was developed by William T. Powers, a physicist/engineer, in the 1950s. He first published it in [68], then formalized it in [66], and revised it in his latest work [32]. According to PCT, through some principles, behavior is defined as (merely) the control of perception: (1) negative feedback leads to control; (2) a specific hierarchical organization of loops leads to control; (3) perception can be only controlled by individuals themselves; (4) conflicts can be caused by controlling others; (5) “dysfunction” can be caused by conflicts between high-level control systems; (6) a specific learning mechanism helps reorganization reestablishes control.”

Moreover, PCT states that control systems are organized in a hierarchy to manage complex goals such as controlling low-level motor as well as regulating high-level psychological and social behavior, by defining the reference signal for the layer below in each layer [66]. The levels of hierarchical perceptual control theory hypothesized by Powers are, respectively, 1st-order: intensity; 2nd order: sensation/vector; 3rd-order: configuration; 4th-order: transitions; 5th-order: sequence; 6th-order: relationships; 7th-order: program; 8th-order: principles; 9th-order: system concepts.

In addition, Powers explained how to generate emotions through a PCT model in his paper [69]: (1) as the brain regulates, the neurochemical reference signals sent from the hypothalamus through the pituitary gland to all major organ systems, and emotion is defined as a product of brain activity; (2) as perceivable changes of physiological state result from disturbances calling control systems into action, emotion is a direct response to the disturbance, the presence of which can be known of instantly by one's conscious awareness; (3) in closed-loop terms, an experienced emotion is caused by “feelings” which is a collection of inputs and perceptions; meanwhile, it outputs a change in the physiological state (e.g., vasoconstriction, respiration rate, metabolism, heart rate, and motor preparedness); (4) an emotion is caused to happen by a reference signal in some high-level system specifying more or less intended amount of some perception, but not by the external factors; (5) in a high-level control system, a zero error signal results from that the perceived current state matches the specified reference signal; while the mismatch will cause a nonzero error signal, so action needs to be taken to correct the error causing emotion; (6) emotional behavior and emotional thinking can be caused by an error signal immediately resulting from a change of reference signal or a change of a disturbance; (7) the strongest negative emotions are related to the largest errors and errors that human beings think need to be corrected most, and when some internal or external factors prevent us from taking action to correct errors, their maximum intensity and duration will appear; (8) when the degree of error is significant and important to them, human beings will use emotional words, leading to awareness of the cause, while small errors mean not using emotional words, leading to failure to identify the cause.

To summarize, emotions are defined to be one aspect of the wholly integrated hierarchy of control by PCT on emotions. The PCT on emotions involves the notion of “an embodiment” (e.g., emotion is defined as a product of brain activity), “adaptation” (e.g., the “general adaptation syndrome” in the case of attack behavior or avoidance), and “appraisals” (e.g., evaluating the significance of an error signal). Consequently, PCT on emotions is compatible with other theories such as the theory of embodied emotion, evolutionary theories, and cognitive-appraisal theories to some extent.

### 2.5. A General Architecture of Computational Models of Emotion

Marsella et al. [5] argued that a number of component “submodels” integrated into the computational models listed in the “family tree” are not clearly delineated. They proposed that by disassembling “submodels” along appropriate joints, a large number of significant differences between different computational models of emotion can be decomposed into a few design choices.

They then proposed a component model view of appraisal models conceptualizing emotions as a set of linked component models (see Figure 2 in [5]) and the relationships between these components. Terminology associated with each of the component models listed in the appraisal architecture was also introduced: (1) person-environment relationship: the term refers to some expression of the relationship between the agent and its environment, which was introduced by Lazarus [2]; (2) appraisal-derivation model: such a model converts some representations of the relationship between a person and the environment into a set of appraisal variables; (3) appraisal variables: they are a set of specific judgments generated as a result of an appraisal-derivation model, which can be used by an agent to produce different emotional responses; (4) affect-derivation model: the mapping from appraisal variables to affective state is processed in this model, and once a pattern of appraisals has been determined, then accordingly how an individual will react emotionally is also specified in this model; (5) affect-intensity model: in the model, a specific appraisal will result in the strength of the emotional response, which is usually calculated by an intensity equation using a subset of appraisal variables, such as desirability and likelihood; (6) emotion/affect: affect could be a set of discrete emotions, a discrete emotion label, core affect in a continuous dimensional space, or even a combination of these factors; (7) affect-consequent model: this model maps affect (or its antecedents) onto some behavioral or cognitive changes which are determined by the behavior consequent models and cognitive consequent models, respectively. Behavior consequent models summarize how affect (e.g., emotion, feeling, and mood) alters an agent's observable physical behavior such as facial expressions, while cognitive consequent models determine how affect will change the nature or content of cognitive processes such as an agent's beliefs, desires, and intentions, respectively.

Three rather different systems, i.e., EMA [70], ALMA [71], and FLAME [72], were characterized in [5] to highlight the conceptual similarities and differences between emotion models by using a component model view of appraisal models. Marsella et al. [5] argued that the adoption of a component view of the model building can empirically assess the capabilities or validity of alternative algorithms to implement the model and conduct meaningful comparisons (i.e., similarities and differences) between systems.

To sum up, Marsella et al.'s compositional view of model building [5], which lays stress on that emotional models, is often composed of individual “submodels” or “smaller components” that can be matched, mixed, or excluded from any given implementation and is often shared. According to Marsella et al. [5], components may be evaluated and subsequently abandoned or improved due to ongoing evaluations before the final version of the model is designed.

## 3. Classical Computational Models of Emotions for Socially Interactive Robots

### 3.1. Kismet's Cognitive-Affective Architecture

With four perceptual modalities (facial display, body posture, gaze control, and speech), an expressive robot called Kismet [73] was developed by MIT, to explore the nature of social interaction and communication between humans. In other words, insights from psychology and ethology [8] have inspired the extensive computational modeling, to explore the social interaction between caregiver and infant.

In view of the key role of infants in normal social development, in order to implement core primitive social response shown by infants, a cognitive-affective architecture emphasizing interactive and parallel systems of cognition and emotion [6] was designed for Kismet. The architecture (see Figure 58.6 in [8]) mainly contains two parts, one is the cognitive systems which are responsible for drives, attention, perception, and goal arbitration while the other part is the affective processes that include affective appraising incoming events, expressive motor behavior (facial expressions, vocalizations, etc.), and basic emotive responses. Therefore, Kismet's models of emotion interact closely with its cognitive system, affecting the behavior and goal arbitration in the architecture [7].

By combining the basis facial postures, Kismet produces a continuous range of expressions (i.e., five primary emotions (happiness, fear, disgust, sadness, and anger) and three additional ones (excitement, interest, and surprise) of varying intensities. This is achieved through the application of an interpolation-based technique in a three-dimensional, componential affect space consisting of the valence, arousal, and stance axes [74], adapted from Russell's circumplex model (arousal and valence) [75], and resonated well with the work of Smith and Scott [76]. Breazeal [74] enumerated a number of advantages gaining from this affect space, such as making the reception of robot facial expressions clearer since only a single state can be expressed at a time (according to selection), enabling reflecting the nuances of the underlying assessment of the robot's facial expressions, and facilitating smooth trajectories through the affect space.

The importance of building an emotional space that allows smooth transitions between discrete emotions was emphasized by the Kismet project, although it does not compare the believability of the expression of smooth transitions and nonsmooth transitions. Moreover, the Kismet project shows that by using a computational model of emotion, a robot can conduct social interaction with humans apart from arbitrating its internal affective states [77, 78].

### 3.2. WE-4RII's Mental Model

The core of the mental model of a robot called WE-4RII (see Figure 58.10 in [8]) is the emotion model. The dynamics of mental transitions in the WE-4RII mental model can be expressed by equations adopting the equation of motion that describes the movement of objects in dynamics [8].

To express the dynamics of mental transitions, the WE-4RII robot has implemented equations of emotion, mood vector, and equations of need (see [79] for more details). Furthermore, the seven basic emotions defined by Ekman [80] are represented as the emotion vector [79, 81] in a three-dimensional mental space consisting of the pleasantness, activation, and certainty axes. Seven emotions and the expressions corresponding to these seven emotions are mapped into a 3-D mental space, and the regional mapping of WE-4RII's emotions is determined by the emotion vector *E* passing through each region (see Figure 58.11 in [8]).

In summary, the mental model of WE-4RII can be computationally implemented [82], as it implements equations inspired by motion to express the dynamics of mental transitions.

### 3.3. PCT-Based Model PRESENCE

To generate robotic emotional behavior, some researchers have designed some computational structures based on PCT on emotions. For instance, a model called PRESENCE “PREdictive SENsorimtor Control and Emulation” which is based on PCT was developed by Moore [83] to improve the speech-based human-machine interaction. Due to PRESENCE, a system can cater to the needs and attention of a user, while a user can allow for the needs and intentions of the system. According to Moore [83], cooperative and communication behaviors are by-products of recursive hierarchical feedback control structures based on this ensemble model.

Some theories and ideas in domains, such as control, neuroscience, bioscience, and psychology, have laid a foundation for the creation of PRESENCE. These theories and ideas include “perceptual control theory,” [66] “mirror neurons,” [84] “hierarchical temporal memory,” [85] and “emulation mechanisms.” [86] To solve three fundamental constraints (i.e., energy, entropy, and time) that ultimately determine the organism's ability to survive within an evolutionary framework, PRESENCE was originally designed as an integrated and recursive processing architecture. To facilitate efficient behavior and efficient communications, PRESENCE maximizes the achievements of the system or the user in the interactive environment, and it is organized into four layers and is therefore inherently recursively nested and therefore hierarchical in structure.

The PRESENCE has been demonstrated in [83] that a Lego NXT computer model was built by Moore to maximize the synchronization of its own behavior with external sources. The robot can sense external sources such as external sounds, can sense its own sounds, and can generate its own rhythmic behavior. Moore's research shows that PCT not only can be used for explaining emotional behavior but also be used in the prediction of emotional behavior.

### 3.4. iGrace Computational Model of Emotions

The iGrace computational model (see Figure 1 in [87] for more details) was designed to enable a companion robot EmI to have a nonverbal emotional response to the speaker's speech. The iGrace consists of 3 principal parts, i.e., the “input” module, the “emotional interaction” module, and the “expression of emotions” module, which can enable EmI to receive input information, process them, and determine emotional behavior. Saint-Aimé et al. [87] described these three modules as follows.

The 7 uplets of the understanding module (i.e., the act of language, actions “for the child,” concepts “for the child,” tense, coherence, phase, and emotional state), the audio signal, and the video signal are taken into account in the “input” module. As such, this module can represent the interface for data exchange and communication between the emotional interaction module and the understanding module.

With the “emotional interaction” module, iGrace can generate the emotional state of EmI using discourse information given by “input” as well as its internal cognitive state. This module contains 4 submodules, namely moderator, selector of emotional experience, generator of emotional experience, and behavior (see more details in [88, 89]), which produce lists Li of pairs (eemo, C (eemo)) involving in four steps (see Figure 2 in [87]) in which C (eemo) denotes an influence coefficient and eemo denotes an emotional experience.

In the “expression of emotions” module, a list of triplet < tone, posture, facial state> is built to express the emotional state of EmI, in which tone is converted into music notes and postures, and facial expressions of EmI are converted into motor movements.

To sum up, the iGrace computational model has demonstrated that it can be computationally implemented in companion robots such as EmI and the new version of EmI [87]. This might result from that iGrace is an instance of the generic model of emotions GRACE [90]. Furthermore, as compared to other computational models of emotions, such as FLAME [91], Kismet [7], Greta [92], EMA [70], and GALAAD [93], GRACE is the only model that applies the three fundamental theories that characterize an emotional process, namely the appraisal theory, coping theory, and personality theory, according to Saint-Aimé et al. [88].

### 3.5. A Computational System of Emotion xEmotion

To allow an agent (a robot carrier) to respond most appropriately to specific changes in the environment, xEmotion, a computational system of emotion, is designed. According to Kowalczuk et al. [94], implementing the intelligent system of decision-making (ISD) in an autonomous agent or robot can make it operate faster and more efficiently, resulting from the ISD's system of emotions, which can be viewed as an approach based on scheduling variable policies from a control theory perspective. Covering various psychological theories on emotions such as the somatic, evolutionary, and appraisal theories of emotion, xEmotion takes into account specific temporal divisions of emotion and, in particular, considers both long-term changes (e.g., personality changes or emotional disorder) and short-term emotions (e.g., expressions or autonomous changes). Furthermore, xEmotion uses (common/real and private/imaginary/individual) wheels/circles of emotion or the “rainbows” of emotions [95] to interpret and compile emotions.

Kowalczuk et al. use a general scheme (see Figure 3 in [94]) to explain how emotions are used as a scheduling variable in the xEmotion system. It takes approximately five big steps to generate emotions in the scheme, namely impression recognition, discoveries recognition, generating emotion/generating equalia (these two phases are parallel), generating mood, and available reactions. And 6 principal components of xEmotion, i.e., autonomous preemotions, expressive subemotions, expressive subequalia, classic emotion, equalia, or private emotion, and mood are distinguished in [94, 96, 97].

For xEmotion to be computationally implementable in the agent (robotic carrier), Kowalczuk et al. [94] applied fuzzy sets in six principal components of xEmotion in the three phases of an emotion process, namely somatic emotions (or preemotions), appraisal of emotions (including subemotions and emotion), and personal emotions (including subequalia, equalia, and mood). The emotional components and their underlying relationships can be found in [98].

In summary, emotions in xEmotion are used not only as scheduling variables (for decision-making and forming responses or general behavior) but also as adjustment parameters (in the motivation subsystem). Furthermore, interpreting and using emotions as a scheduling control variable have made some contributions to the research and the implementation of the computational model of emotions for robots.

## 4. Our Proposed Computational Model of Robotic Emotions

In this section, we first propose a computational model of emotions for socially interactive robots, especially for robots for a special group of people such as autistic children, so-called AppraisalCloudPCT (based on a component view of computational models, the appraisal theories on emotions, cloud robotics, and perceptual control theory on emotions), then we compare our model AppraisalCloudPCT with the five models for robotic emotions revisited in Section 3.

### 4.1. Our Computational Model AppraisalCloud PCT

#### 4.1.1. Principles in Design of a New Model

There are certain key points we want to stress, or several problems (e.g., how can a robot highly emulate human emotions? how can a computational model of emotions be highly computable? how can a computational model of emotions be suitable for most of the socially interactive robots and be computationally implementable in them?) we want to tackle when designing a new computational model of robotic emotions.

The followings are given five primary principles in designing our new model:

1. Principle in the simulation of human emotions: with a computational model of emotions, a robot may simulate the whole process of a human emotion (e.g., generation, regulation, and responding to a stimulus), and as such, the computational model can highly simulate a human emotion
2. Principle in achieving computability of the model: each component of the model should be computable, and as a whole, the model should be computable
3. Principle in enhancing human-robot interaction: a computational model of emotions should not only take into account a user's intention (or need), attention, emotional state, the response to the robot, and the impact of the external environment (such as noise, disturbance, contextual cues) on the user during the interaction but also coordinate the robot to make an appropriate response to the surrounding emotional environment
4. Principle in promoting the universality of a computational model in socially interactive robots: as more and more socially interactive robots are deployed in therapy and rehabilitation situations, a computational model of emotions should take into account the social and communicative characteristics of a special group of users such as autistic children or dementia elders
5. Principle in facilitating sharing information between and learning from socially interactive robots: a computational model of emotions should endow a robot with a more powerful capability of making decisions faster, more appropriate, and more efficient, given that more and more socially interactive robots will be exposed to various users with different backgrounds and be connected to substantial Internet of Things (IoT) such as medical IoT with massive medical data

#### 4.1.2. An Overview of the New Model Appraisal Cloud PCT

**Figure 1:**
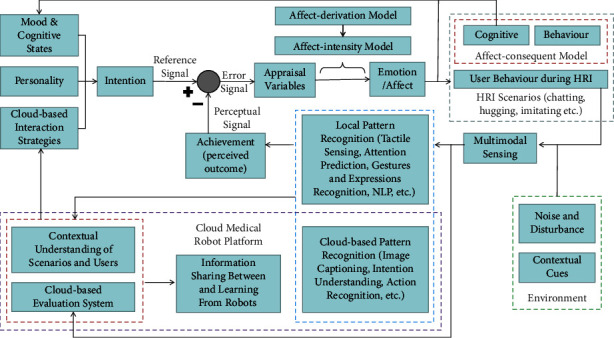
The whole architecture of the proposed model AppraisalCloudPCT.

Based on the five primary principles in designing a model mentioned previously, we designed a new computational model of robotic emotions AppraisalCloudPCT as illustrated in Figure 1.

The theoretical basis and guiding methodology covered in the proposed model in response to each of the 5 primary principles are introduced as follows:

1. The proposed computational model adopts the concepts of perceptual control theory (PCT) on emotions [32] and PCT-based PRESENCE [83] to achieve simulation of human emotions: in a closed-loop as illustrated in Figure 1, a collection of the intention of a robot (i.e., a reference signal) and achievement (perceived outcome) (i.e., a perceptual signal) will cause an experienced emotion, and at the same time, an output-caused change in the cognitive states and behavior of the robot will affect a user's behavior during the human-robot interaction. In other words, the difference (i.e., a mismatch) between the reference signal and the perceptual signal will immediately result in an error signal, which will give rise both to the emotional behavior and to the emotional thinking of a robot. And emotions with greater intensity and longer duration will arise in connection with a larger error that demands a robot to alter its affect-consequent model more appropriately to correct the error. Moreover, with the computational model, a robot will be endowed with mood and cognitive states, personality, and cloud-based interaction strategies to form its intention. As such, the computational model can highly simulate the whole process (e.g., intention, generation, regulation, and responding to a stimulus) of a human emotion.
2. The proposed computational model adopts Marsella et al.‘s compositional view of model building [5], which lays stress on that emotional models are often composed of individual “submodels” or “smaller components” that can be matched, mixed, or excluded from any given implementation and are often shared. And 5 out of 7 component models listed in the appraisal architecture in [5] are adopted in our proposed computational model, namely appraisal variables, affect-derivation model, affect-intensity model, emotion/affect, affect-consequent model consisting of the cognitive-consequent model, and behavior-consequent model. As illustrated in Figure 1, our proposed model is assembled from more than 15 “submodels.” Consequently, when each of them is computable, the computability of our proposed model as a whole can be achieved.
3. On one hand, to make human-robot interaction more effective, efficient, or pleasant, the achievement (perceived outcome), e.g., the interpretation of a user's intention, attention, emotional state, and behavior, will influence the appraisal variables in the proposed computational model. On the other hand, to coordinate a robot to respond to the surrounding emotional contexts (i.e., contextual cues in the environment containing emotional information that might have an impact on a user's interpretation of the behavior of a robot [99–102]) appropriately, so that the robot can fit with its environment better, contextual understanding of the scenarios and the user is taken into account to support the cloud-based interaction strategies in the proposed computational model.
4. The proposed computational model of emotions takes into account the social and communicative characteristics of a special group of users such as autistic children or dementia elders, through its submodel so-called cloud-based interaction strategies, which is supported by two submodels (i.e., “contextual understanding of scenarios and users” and “cloud-based evaluation system”) in a cloud medical robot platform, as illustrated in Figure 1. A cloud-based evaluation system may have certain advantages as mentioned in the research on cloud medical robots [35–38], one of which is data of interaction between a user and a robot can be stored and evaluated in a cloud for further assessment of the social and communicative characteristics of a user. And the submodel “contextual understanding of scenarios and users” relies on another two submodels “local pattern recognition” and “cloud-based pattern recognition,” which can provide the interpretation of a user's intention, attention, emotional state, and behavior. Therefore, the proposed computational model of emotions is suitable for socially interactive robots, especially for robots for a special group of users such as autistic children or dementia elders, which promotes the universality of our model to some extent.
5. To facilitate sharing information between and learning from socially interactive robots, a cloud medical robot platform is built and assembled in the proposed computational model. With such a platform, information can be shared between robots through the submodel “cloud-based evaluation system,” and the capability of interpretation of a user and of making decisions can be learned through the submodel “contextual understanding of scenarios and users.”

### 4.2. Comparison of Models

This section compares the 5 computational models for robotic emotions revisited in Section 3 with our proposed computational model (see Table 1 for a summary). The five crucial properties of a computational emotion model, (i) domain-independent, (ii) models mood, (iii) models personality, (iv) data-driven mapping, and (v) ethical reasoning, as listed in a review paper [103], alongside with one more property (vi) combining with cloud robotics (we believe this will be a future trend in building the computational models of emotions for socially interactive robots), are chosen as the six criteria for comparison.

Table 1 shows a comparative assessment between the computational models of emotions for socially interactive robots as can be inferred from the summary in the table, even to satisfy the first five criteria still remains as a challenge. Great efforts have been made in building our proposed computational model of emotions to meet all the six criteria, by adopting the appraisal theories on emotions, perceptual control theory on emotions, a component model view of appraisal models, and cloud robotics. How our proposed computational model meet all the six criteria is summarized as follows: (1) to meet of the criteria of “domain-independent,” our proposed computational model not only takes into account the social and communicative characteristics of every user but also can coordinate a robot implementing our model to respond to the surrounding emotional contexts appropriately; (2) mood is considered as a long-term change in a submodel “mood and cognitive states” of our proposed computational model, and it is impacted by the other two submodels “emotion/affect” and “cognitive,” and therefore, the second criteria “models mood” can be met; (3) there is a submodel “personality” in our proposed computational model such that personality can be modeled; (4) between appraisal variables and emotions, there are two consecutive submodels “affect-derivation model” and “affect-intensity model” in our proposed computational model, which supports data-driven mapping of the appraisal variables into emotion intensities; (5) a emotion regulation mechanism is implemented in our proposed computational model through a closed-loop emotion modeling and regulation based on perceptual control theory on emotions, and through a submodel “cloud-based interaction strategies,” (6) our proposed computational model combines with cloud robotics by using a submodel “cloud medical robot platform.”

## 5. The Implementation of Our Model in a Social Robot for Autistic Rehabilitation

### 5.1. A Social Robot for Autistic Rehabilitation

**Figure 2:**
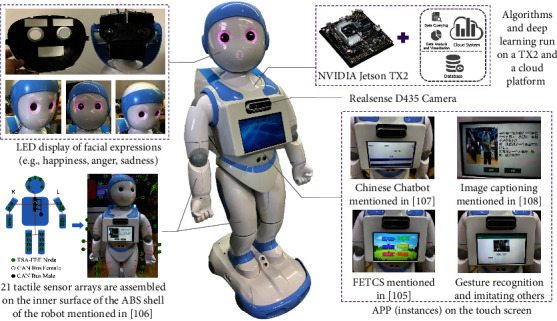
The appearance and functionalities of our developed robot Dabao for autistic rehabilitation.

**Figure 3:**
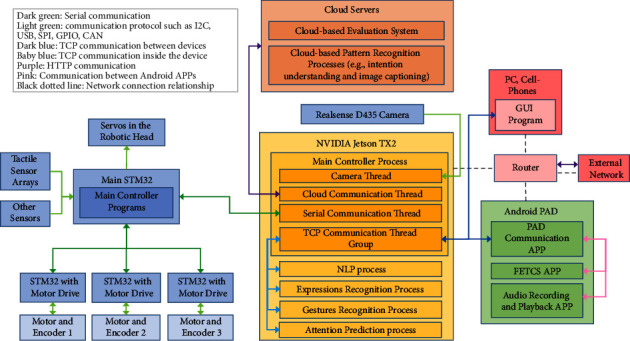
The software architecture of the robot.

We developed a socially interactive robot so-called Dabao for autistic rehabilitation, with which we conducted three preliminary clinical human-robot interaction studies [10, 104, 105] for Chinese children with ASD. The appearance and functionalities of the robot are demonstrated in Figure 2, and the software architecture is illustrated in Figure 3 as follows.

**Figure 4:**
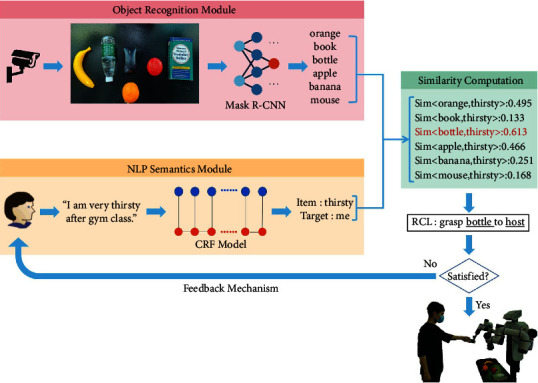
A new task-based framework that enables robots to understand human intentions using visual-NLP semantic information [109]: it includes a language semantics module to extract keywords no matter if the command directive is explicit or not, a visual object recognition module to identify multiple objects located to the front of the robot, and a similarity computation algorithm for inferring the intention based on a given task (i.e., selecting some desired item out of multiple objects on a table and giving it to a particular user among several human participants). Result of the similarity computation is then translated into structured robot control language RCL (grasp object to place) to be comprehended by robots. The experimental results demonstrate the ability of the framework to allow robots to grasp objects with the actual intent of vague, feeling, and clear type instructions.

**Figure 5:**
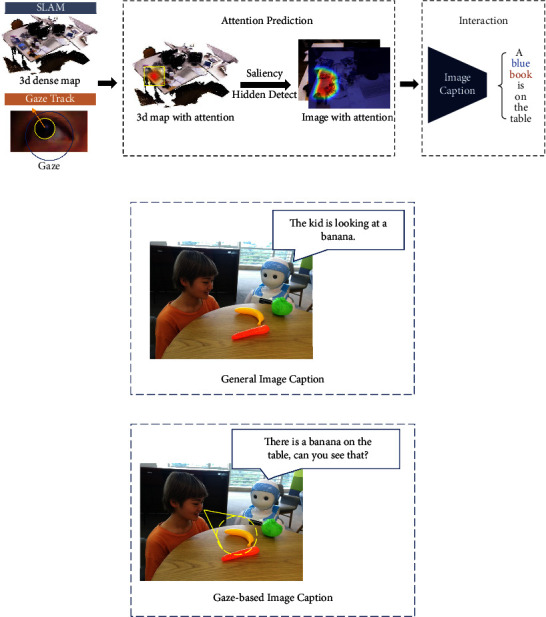
The overall framework of a novel gaze-based image caption system for autistic children and the effect of the framework in a gaze-based image caption system [110]: (a) the overall framework describes the region where an autistic child is looking at and combines image caption (based on attention heat maps, it describes the region concentrated by the child) with gaze-following (it is based on spatial geometry and predicts areas of attention from the spatial relationship between the map and line of sight); (c) is more suitable than (b) in enhancing human-robot interaction and in promoting the spontaneous language development of autistic children, as adding gaze-following can support a robot in better describing what the child is looking at.

Apart from the tactile sensing [106] and some APP instances [105, 107, 108] on the touch screen as demonstrated in Figure 2, we have developed some other deep learning algorithms to endow the robot with a stronger capability in the interpretation of a user (e.g., an autistic child), such as intention understanding (see Figure 4) and attention recognition (see Figure 5). Furthermore, Table 2 summarizes six major capabilities of the robot to perceive a user, to infer a user's mood and cognitive states and behavior, and to express itself to a user that can influence the effect, the efficiency, and the pleasantness in the human-robot interaction.

### 5.2. The Implementation of Our Model in the Social Robot

**Figure 6:**
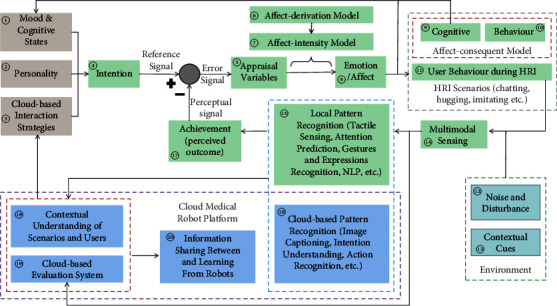
Compositional submodels (marked in red circled numeric symbol) of our proposed model AppraisalCloudPCT: (1) the submodels (i.e., number 4–17) marked in the green color theme constitute the main recursive control loop of a robot's processing of emotional information, adopting perceptual control theory on emotions and a component model view of appraisal models; (2) the submodels (i.e., number 1–3) marked in the yellow-brown color theme constitute a robot's intention of how to appraise an event (i.e., appraisal patterns of an interaction process), in which the appraisal patterns of the nine appraisal dimensions of a robot's emotion can be affected by a robot's mood and personality, and the interaction strategies; (3) the submodels (i.e., number 16–20) marked in the green color theme constitute the “cloud medical robot platform”; (4) the submodels (i.e., number 12-13) marked in the cyan color theme indicate that a robot will not only take the impact of the external environment (such as noise, disturbance, contextual cues) on the user during the interaction into account but also respond to the surrounding contexts appropriately.

As illustrated in Figure 6 (as equivalent to Figure 1, except for all of the submodels are marked in different numeric symbols and different color themes, for a better explanation of how our model is implemented in the social robot Dabao developed by us), our proposed model AppraisalCloudPCT consists of 20 compositional submodels (or so-called components of a model). Such a compositional view of the model building has certain advantages, one of which is that we can implement the proposed model AppraisalCloudPCT in our social robot by implementing its compositional submodels one by one and then by forming the whole model in a closed-loop control.

We implement each submodel with mathematical definitions and formulas in our social robot as follows.

#### 5.2.1. The 1^st^ Submodel “Mood and Cognitive States”

The equation of mood, equation of emotion, as defined in [112], will be adopted in implementing our proposed model AppraisalCloudPCT. First, emotion vector *E* can be defined in the PAD mental space consisting of the pleasantness, arousal, and dominance axes as the robot's cognitive state as follows:

(1) $$\begin{array}{c} E=\left(E_{p},E_{a},E_{d}\right)\text{,} \end{array}$$

where *E*_*p*_ is the pleasantness component of emotion, *E*_*a*_ is the arousal component of emotion, and Ed is the dominance component of emotion.

According to Itoh et al. [112], mood vector *M*, consisting of a pleasantness component and an arousal component, can be defined as follows:

(2) $$\begin{array}{c} M=\left(M_{p},M_{a},0\right)\text{,} \end{array}$$

(3) $$\begin{array}{c} M_{p}=\int E_{p}dt\text{,} \end{array}$$

(4) $$\begin{array}{c} {\ddot{M}}_{a}+\left(1-M_{a}^{2}\right){\dot{M}}_{a}+M_{a}\text{,} \end{array}$$

where *M*_*p*_ and *M*_*a*_ denote the pleasantness and arousal components of the mood, respectively. The integral of the pleasantness component of the emotion equation (3) defines *M*_*p*_, resulting from that the pleasantness of mood can be influenced by the current cognitive state. Furthermore, *M*_*a*_ has been defined by the Van del Pol equation (4) owing to that the activation component of mood vector is similar to the biological rhythm of the human body, such as the internal clock.

#### 5.2.2. The 2nd Submodel “Personality”

By far, the big five personality traits (i.e., openness (*O*), conscientiousness (*C*), extraversion (*E*), agreeableness (*A*), and neuroticism (*N*)), as defined in [113, 114]), were the most widely used measure for human and robot personality modeling in human-robot interaction literature. Three main conclusions can be drawn from the literature review in [115]: (1) extroverts seemingly react more positively in the period of interaction with robots; (2) humans respond more positively to extroverted robots, but this relationship is moderate; (3) humans respond well to robots with similar and/or different personalities. Furthermore, Robert [115] suggested the effects of context on the impact of robot and human personality to be looked at in future studies, as it is easy speculating that the personality of a robot may be more important to a home robot rather than one used at work. This is consistent with the contextual approach to personality, whereby a person's personality is best described and understood in the various contexts in which it is placed [116]. Moreover, the users' preferences for robot personalities can be determined by people's stereotype perceptions of certain jobs and the background of the robot's role [117]. Therefore, the behavior of the robot may need to be adapted to the user's expectations as to what personality and behavior are consistent with such tasks or roles.

In a recent study [118], researchers found that participants performed better when using a robotic assistant with a similar personality to their own or a human assistant with a different personality. This is in accordance with the results of the systematic evaluation of human and robot personality in healthcare human-robot interaction [119] that matching the patient and robot personality based on introversion or extroversion is positively correlated with beneficial results. Research in [119] also found that robot personality traits such as extroverted, feminine, responsive, amiability, and sociable were positively associated with beneficial outcomes.

Not only the emotional factors [120] but also the appraisal patterns of emotion [121] can be affected by the Big Five personality traits. The relationship between the PAD model [41] and the five factors of personality can be derived through the linear regression analysis in [120]. And three equations of temperament including pleasure, arousal, and dominance are summarized in [122] as follows:

(5) $$\begin{array}{c} P\alpha =0.21E+0.59A+0.19N\text{,}\\ P\beta =0.15O+0.30A-0.57N\text{,}\\ P\gamma =0.25O+0.17C+0.60E-0.32A\text{,} \end{array}$$

where *Pα* denotes the value for the pleasant axis (*α*-axis), *Pβ* denotes the value for the arousal axis (*β*-axis), and *Pγ* denotes the value for the dominance axis (*γ*-axis), respectively. Furthermore, the five factors of personality, i.e., *O*, *C*, *E*, *A*, *N* ∈ [−1, 1], where *O* for openness, *C* for conscientiousness, *E* for extraversion, *A* for agreeableness, and *N* for neuroticism, respectively.

The relationships between the five factors of personality and the appraisal dimensions of emotion could be derived in [121] (Page 519), where 10 main appraisal dimensions in major appraisal theories (Pleasantness, Goal Conduciveness, Effort, Perceived Control, Certainty, Agency-Self, Agency-Others, Agency-Circumstances, Unfairness, and Moral Violation), plus a new appraisal, relationship-involvement, were selected (see the Appendix in [121] for more details). Similarly, 9 personality-appraisal relationships (no relationship was found for appraisals “effort” and “relationship-involvement”) in [121] (Page 519) can be summarized as follows:

(6) $$\begin{array}{c} Pleasantness\left(F_{pl}\right)=-0.585N+0.606C\text{,} \end{array}$$

(7) $$\begin{array}{c} Goal-Condu\;civeness\left(F_{gc}\right)=-0.579N+0.369C\text{,} \end{array}$$

(8) $$\begin{array}{c} Perceived\;Control\left(F_{pc}\right)=-1.281N+0.923E+1.306C\text{,} \end{array}$$

(9) $$\begin{array}{c} Certainty\left(F_{c}\right)=-1.203N+0.880C\text{,} \end{array}$$

(10) $$\begin{array}{c} Agency-Self\left(F_{as}\right)=-0.808A\text{,} \end{array}$$

(11) $$\begin{array}{c} Agency-Others\left(F_{ao}\right)=-0.965C+0.950O\text{,} \end{array}$$

(12) $$\begin{array}{c} Agency-Circumstances\left(F_{ac}\right)=-0.587C\text{,} \end{array}$$

(13) $$\begin{array}{c} Unfairness\left(F_{u}\right)=1.149N-0.928E-1.113C\text{,} \end{array}$$

(14) $$\begin{array}{c} Moral\;Violation\left(F_{mv}\right)=1.309N-1.005E-1.456C-0.840O\text{,} \end{array}$$

where *O*, *C*, *E*, *A*, *N* ∈ [−1, 1]

Each equation indicates a relationship between an appraisal dimension and a combination of the Big Five personality traits, i.e., the tendency of appraising events in the particular appraisal dimension by people with specific personality traits. For instance, in equations (6) and (7), people with low *N* and high *C* will be more likely to appraise events as pleasant (Pleasantness) and as conducive to important goals (Goal-Conduciveness), although the tendency of appraising the same event in the two appraisal dimensions is not exactly the same. Note that once the value of the Big Five personality traits is determined, the value of each appraisal dimension will be also determined in equations (6)–(14).

#### 5.2.3. The 3^rd^ Submodel “Cloud-Based Interaction Strategies”

The main purpose of this submodel is to output a strategy that a robot can use in the next round of interaction with an autistic child. Adopting the perceptual control theory on emotions, our proposed model AppraisalCloudPCT is designed in the first place to enable many rounds of recursive interaction between a robot and an autistic child, so that the interaction will be more effective, efficient, and easier to be satisfied by the child. By “strategy,” it means that, given the specific estimation of valence, arousal, and engagement levels of the child supported by the submodel “cloud-based evaluation system” and the contextual understanding of the interactive scenario and the child supported by the submodel “contextual understanding of scenarios and users,” the robot will be able to alter its mood and personality to match with the status of the child and the interactive context, for a better round of interaction.

**Figure 7:**
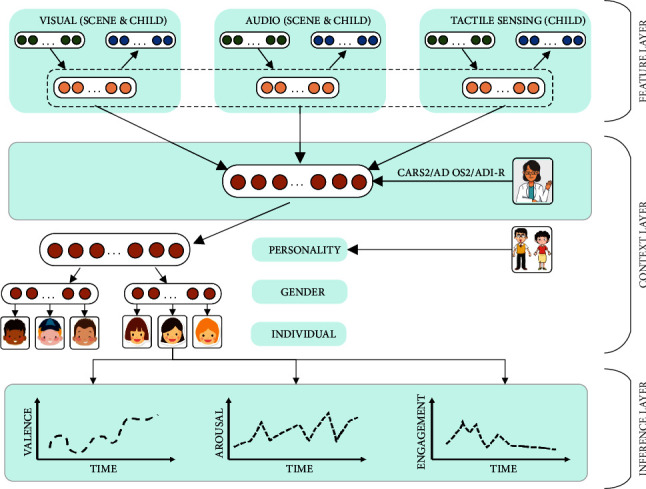
Three layers of modified PPA-net based on the work in [123]: (1) feature fusion is performed in the feature layer using features from three modalities (visual, audio, and tactile); (2) the context layer firstly uses behavioral scores of the child's verbal ability, motor, and mental, to augment the input features using the autistic rating scales such as CARS2 [124], ADOS-2 [125], and ADI-R [126], and then, the GPA-NET (group-level network) is trained and used to initialize the personalized PPA-net weights at the personality, gender, and individual level (using clone); (3) the third layer is the inference layer, in which the child-specific estimation of valence, arousal, and engagement levels will be performed.

As mentioned above in 5.2.2, for a better performance in human-robot interaction, a robot should have a similar personality to human participants, and the effects of context should be taken into consideration when designing a robotic personality. In this study, it is, therefore, necessary for the robot to have knowledge of the personality profile of an autistic child (this can be supported by the 19th submodel “cloud-based evaluation system,” as illustrated in Figure 7 that personality profile can be provided by the child's parents) and to understand the interactive scenario and the child in the surrounding context (this can be supported by the 18th submodel “contextual understanding of scenarios and users,” please refer to it for more details).

Consequently, this submodel will output cloud-based interaction strategies as follows:

  Strategy one: To match a robot's personality with that of a child, first, the personality profile (i.e., rating scales of *O*, *C*, *E*, *A*, *N* between −1 and 1) of an autistic child who will interact with the robot will be obtained, and then, a robot's personality will match with the child's personality. Once the personality of the robot is altered, the emotional tendency that the robot will be experiencing and the appraisal patterns that the robot will use can be predicted by using the (10) equations in 5.2.1 and 5.2.2.
  Strategy two: As contexts effect of a user's perception of not only the emotions but also the personality of a robot, effects of context should be taken into consideration. First, the role that the robot plays in the task of the HRI scenario and what kind of personality that an autistic child expects to be consistent with such a task or role should be identified in the first place. Then, the personality of the robot should be modified to adapt to the child's expectation.
  Strategy three: The outcome of “contextual understanding of scenarios and users” should be taken into account, given that the noise and disturbance, and contextual cues may influence an autistic child's mood and his/her judgement of the robot's emotions. To do that, first, the emotional valence of the contextual cues will be obtained. Then, the robot's mood should be congruent with the emotional valence of the contextual cues to some extent. Thirdly, in case of noise and disturbance were detected in the HRI scenario, the robot's estimation of the child's valence and arousal levels provided by the submodel “cloud-based evaluation system” should be rectified to some extent depending on the amount of the noise and disturbance.

#### 5.2.4. The 4th Submodel “Intention”

“Intention” in this submodel means how will a robot intends to appraise an event (i.e., appraisal patterns of an interaction process), based on a robot's mood and personality with the consideration of an interaction strategy for the next round of interaction. The main purpose of this submodel is to map the outputs of the first three submodels, namely, “mood and cognitive states,” “personality,” “cloud-based interaction strategies,” into appraisal patterns, which can be defined as follows:

(15) $$\begin{array}{c} F_{intention}=\left(F_{pl}+∆pl,F_{gc}+∆gc,F_{pc}+∆pc,Fc+∆c,F_{as}+∆as,F_{ao}+∆ao,F_{ac}+∆ac,Fu+∆u,F_{mv}+∆mv\right)\text{,} \end{array}$$

where *F*_pl_, *F*_gc_, *F*_pc_, *F*_*c*_, *F*_as_, *F*_ao_, *F*_ac_, *Fu*, *F*_mv_ represent Pleasantness, Goal Conduciveness, Perceived Control, Certainty, Agency-Self, Agency-Others, Agency-Circumstances, Unfairness, and Moral Violation, respectively, as defined in Equation (6)–(14) in 5.2.2, and ∆pl, ∆gc, ∆pc, ∆*c*, ∆as, ∆ao, ∆ac, ∆u, ∆mv represent the impact of the two submodels “mood and cognitive states” and “cloud-based interaction strategies” on the tendency of appraising events in the particular appraisal dimension, respectively.

#### 5.2.5. The 5^th^ Submodel “Appraisal Variables”

As mentioned in 4.1.2, in a closed-loop as illustrated in Figure 1, a collection of intention of a robot (i.e., a reference signal) and achievement (perceived outcome) (i.e., a perceptual signal) will cause an experienced emotion, and at the same time, an output-caused change in the cognitive states and behavior of the robot will affect a user's behavior during the human-robot interaction. In other words, the difference (i.e., a mismatch) between the reference signal and the perceptual signal will immediately result in an error signal, which will give rise both to emotional behavior and thinking of a robot.

Appraisal variables are defined as the set of specific judgments by which a robot can generate different emotional responses. The main purpose of this submodel is to output the error signal (i.e., a mismatch between a collection of intention of the robot and the achievement (perceived outcome)) as appraisal variables. Here, the error signal can be defined as follows:

(16) $$\begin{array}{c} F_{error}=F_{intention}-F_{perceived}\text{,} \end{array}$$

where *F*_intention_ represents a collection of intention of the robot as defined in equation (15) in 5.2.4, and Fperceived represents the achievement (perceived outcome) of the robot as defined in equation (18) in 5.2.17.

#### 5.2.6. The 6^th^ Submodel “Affect-Derivation Model”

This submodel will specify the mapping from appraisal variables to affective state, and once a pattern of appraisals has been determined how a robot will react emotionally. According to Itoh et al. [112], emotion vector *E* = (*E*_*p*_, *E*_*a*_, *E*_*d*_) can be expanded into the second-order differential equation as shown in equation (17) as follows:

(17) $$\begin{array}{c} M\ddot{E}+\Gamma \ddot{E}+KE=F_{EA}\text{,} \end{array}$$

where *M*, Γ, *K*, *F*_EA_ represent the emotional inertia matrix, emotional viscosity matrix, emotional elasticity matrix, and emotional appraisal, respectively. And the emotional appraisal FEA stands for the total result of appraising the appraisal variables (i.e., the error signal Ferror). According to Itoh et al. [112], by changing the emotional coefficient matrixes, the robot can express different reactions to a same stimulus.

#### 5.2.7. The 7^th^ Submodel “Affect-Intensity Model”

The strength of the emotional response resulting from a specific appraisal is specified in this submodel. As mentioned in 4.1.2, emotions with greater intensity and longer duration will arise in connection with a larger error that demands a robot to alter its affect-consequent model more appropriately to correct the error. Therefore, the bigger the error signal Ferror becomes, the greater the intensity of and with longer duration an emotion will be, and the stronger the emotional response will be.

#### 5.2.8. The 8th Submodel “Emotion/Affect”

For each discrete emotion the robot will be experiencing, emotion vector *E* = (*E*_*p*_, *E*_*a*_, *E*_*d*_) can be mapped in the PAD mental space consisting of the pleasantness, arousal, and dominance axes. For mood vector, *M* = (*M*_*p*_, *M*_*a*_, 0) consisting of a pleasantness component *M*_*p*_ and an arousal component *M*_*a*_ can also be mapped in the PAD mental space.

#### 5.2.9. The 9^th^ Submodel “Cognitive-Consequent Model”

This submodel determines how affect alters the nature or content of cognitive processes such as a robot's beliefs, desires, and intentions, respectively. As mentioned above, an error signal (i.e., a mismatch between the intention of the robot and the achievement (perceived outcome)) will result in a robot's intention to correct the error. How strong will the intention to correct the error be depends on how big the error is. Furthermore, as the robot is experiencing an emotion, its mood will be effected to some extent.

#### 5.2.10. The 10^th^ Submodel “Behavior-Consequent Model”

This submodel summarizes how affect alters our robot's observable physical behavior such as facial expressions. As described in Table 2 in Section 5.1, our robot is equipped with 6 key capabilities in interactive scenarios with Chinese autistic children, and it can express itself to the children through facial expressions, gestures, speech, etc. In the interactive scenarios, the robot should alter its observable physical behavior in a manner, according to not only the emotion it is experiencing but also the three cloud-based interaction strategies described in 5.2.3.

#### 5.2.11. The 11^th^ Submodel “User Behavior during HRI”

In the child-robot interaction scenarios (e.g., having a conversation, hugging, playing games), an autistic child will generate certain behavior to adopt to/finish/withdraw from the child-robot interaction. Such behavior (e.g., gaze regulation, facial expressions, hand and body gestures, verbal expression), not only is a product of the child-robot interaction but also can be effected by the outward behavior of the robot as defined in the submodel “behavior-consequent model.”

#### 5.2.12. The 12^th^ Submodel “Noise and Disturbance”

Noise in this submodel is defined as noise coming from the surrounding contexts (e.g., ambient noise, other human voices other than the voice of the autistic child during the child-robot conversation). And disturbance is defined as any unexpected event that will have an adverse impact on the child-robot conversation, such as a heavy push on the robot, and the autistic child is forced by somebody to end the child-robot interaction in advance. Both the noise and disturbance can be detected by the sensors to perceive the child and the environment and by the self-checking sensors (e.g., torque sensors) inside the robot.

#### 5.2.13. The 13^th^ Submodel “Contextual Cues”

Robot faces can be viewed in the same way as human faces, according to [102] that users' perceptions of a robot's simulated emotional expressions can be affected by different emotional surrounding contexts (i.e., consistent or inconsistent classical music, or BBC news). Furthermore, when there is emotional context around, people are more able to recognize the facial expressions of the robot when the emotional valence of the environment is consistent with the facial expressions of the robot than when the emotional valence of the environment and its facial expressions are not consistent [99–101].

Consequently, it is important for the robot to perceive the emotional valence (i.e., contextual cues) of the surrounding contexts (e.g., sound, music, pictures/posters on the wall, video clips on the TV) in the interactive scenarios. As such, contextual cues will be considered, collected, and added to our proposed model AppraisalCloudPCT in this submodel.

#### 5.2.14. The 14^th^ Submodel “Multimodal Sensing”

In this submodel, the robot will perceive the autistic child and sense the environment through various sensors (e.g., camera, microphone arrays, tactile sensing arrays, infrared sensor) and multiple modalities (e.g., visual, auditory, and tactile sensing). A collection of sensor data in this submodel will feed to two submodels “local pattern recognition” and “cloud-based pattern recognition” and will be uploaded to the cloud medical robot platform, more specifically, to the submodel “cloud-based evaluation system.”

#### 5.2.15. The 15^th^ Submodel “Local Pattern Recognition”

In this submodel, our proposed model AppraisalCloudPCT will output the results of the local pattern recognition (i.e., processes that run on the NVIDIA Jetson TX2 inside the robot body, as illustrated in Figure 3), and the results will uploaded to the cloud medical robot platform to facilitate the two submodels, i.e., the cloud-based evaluation system and the contextual understanding of scenarios and users.

The output of tactile sensing is defined as TS = (*P*_*i*_, TB_*j*_), where *P*_*i*_ is the *i*th position of the robot body being touched by an autistic child *P*_*i*_ ∈ {Top of Head, Back of Head, Forehead, Left Cheek, Right Cheek, Front of Right Forearm, Back of Right Forearm, Front of Right Upper Arm, Back of Right Upper Arm, Back of Left Upper Arm, Front of Left Upper Arm, Back of Left Forearm, Front of Left Forearm, Right Rear Hip, Back of Right Thigh, Inner Right Thigh, Lower Right Thigh, Left Rear Hip, Back of Left Thigh, Inner Left Thigh, Lower Left Thigh}, and TB_*j*_ is the *j*th touching behavior pattern of the autistic child TB_*j*_ ∈ {Palm Momentary Sliding, Palm Momentary Tapping, Random Finger Poking, Finger Sliding, Random Slow Sliding, Random Momentary Tapping}.

The output of Attention Prediction (i.e., gaze and head direction estimation) is defined as AP = (dl, dr, dh), where dl and dr are gaze direction of the left and right eyes of an autistic child respectively, and parameter dh represents the head direction.

The output of gestures recognition is defined as GR = (HG_*i*_, BG_*j*_), where HG_*i*_ is the hand gesture of an autistic child HG_*i*_ ∈ {OK, Peace, Punch, Stop, Nothing}, and BG_*j*_ is the body gesture of the autistic child BG_*j*_ ∈ {Standing, Walking, Running, Jumping, Sitting, Squatting, Kicking, Punching, Waving, None}.

The output of facial expressions recognition is defined as FE_*i*_ ∈ {Happiness, Sadness, Anger, Surprise, Fear, Disgust, Neutral}.

The output of natural language processing (NLP) is not defined as the whole sentences in a conversation between the robot and an autistic child, but as pertinent words or word stems in natural languages that can commonly distinguish 36 affective categories, as defined in [57] (Page 714–715). Therefore, NLPoutput = (PWs, AC), where PWs represents all of the pertinent words or word stems as defined in [57] that can be extracted from a conversation and affective category AC ∈ {Contentment, Anger, Admiration/Awe, Anxiety, Amusement, Being, Touched, Desperation, Boredom, Compassion, Contempt, Disappointment, Disgust, Dissatisfaction, Envy, Fear, Feeling, Gratitude, Guilt, Happiness, Hatred, Hope, Humility, Interest/Enthusiasm, Irritation, Jealousy, Joy, Longing, Lust, Pleasure/Enjoyment, Pride, Relief, Sadness, Relaxation/Serenity, Tension/Stress, Shame, Surprise, Positive, Negative, Neutral} (36 affective categories plus Neutral).

#### 5.2.16. The 16^th^ Submodel “Cloud-Based Pattern Recognition”

In this submodel, our proposed model AppraisalCloudPCT will output the results of the cloud-based pattern recognition (i.e., processes that run on the cloud, as illustrated in Figure 3), and the results will be uploaded to the cloud medical robot platform to facilitate the three submodels, i.e., the cloud-based evaluation system, the contextual understanding of scenarios and users, and the information sharing between and learning from robots.

The output of image captioning is defined as IC = (Ob, Pr, At), where Ob represents the object concentrated by an autistic child, the region of which can be represented by an attention heat map of an image captured by the robot camera, Pr represents the preposition, and At represents the attributes of the object.

The output of intention understanding is defined as IU = (Insi, TO, DP, RCL), where Insi is one of the three types of natural language instructions given by an autistic child Insi ∈ {Clear Type, Vague Type, Feeling Type}, TO represents the target object out of multiple objects in front of the robot, DP represents the delivery place that the target object should be delivered to, and the RCL format utilized in this paper is “Grasp TO to DP,” which is the structured language that can be comprehended by robots.

The output of action recognition is defined as AR = (AB_*i*_, HB_*j*_), where AB_*i*_ belongs to 10 kinds of abnormal behaviors of an autistic child plus the normal status AB*i* ∈ {Clapping Hands, Swinging Back and Forth, Spinning Circles, Flipping Fingers, Bumping Heads, Clapping Ears, Turning Fingers, Scratching, Walking on Tiptoe, Snapping Fingers, Normal Status} and HB_*j*_ belongs to 5 kinds of unhealthy conditions plus the healthy status HB_*j*_ ∈ {Falling Down, Having Headache, Having Chest and Abdominal Pain, Having Back Pain, Having Neck Pain, Healthy Status}.

#### 5.2.17. The 17^th^ Submodel “Achievement (Perceived Outcome)”

The importance of this submodel is to summarize the feedback (e.g., the interpretation of an autistic child's intention, attention, emotional state, and behavior) provided by the child during/after the human-robot interaction. The achievement (perceived outcome) of the robot can be defined as follows:

(18) $$\begin{array}{c} F_{perceived}=\left({PF}_{pl},{PF}_{gc},{PF}_{pc},{PF}_{c},{PF}_{as},{PF}_{ao},{PF}_{ac},{PF}_{u},{PF}_{mv}\right)\text{,} \end{array}$$

where *PF*_*pl*_, *PF*_*gc*_, PF_pc_, PF_*c*_, PF_as_, PF_ao_, PF_ac_, *PF*_*u*_, *PF*_*mv*_ represent the 9 appraisal dimensions respectively as described in 5.2.2, i.e., Pleasantness, Goal Conduciveness, Perceived Control, Certainty, Agency-Self, Agency-Others, Agency-Circumstances, Unfairness, and Moral Violation that will be used to appraise the achievement (perceived outcome). These 9 appraisal dimensions will be defined in equation (19)–with *PF*_*pl*_ as follows:

(19) $$\begin{array}{c} {PF}_{pl}=a_{1}\cdot {OT}_{1}+a_{2}\cdot {OT}_{2}+a_{3}\cdot {OT}_{3}+a_{4}\cdot {OT}_{4}+a_{5}\cdot {OT}_{5}\text{,} \end{array}$$

where OT_1_, OT_2_, OT_3_, OT_4_ ∈ [−1, 1], and OT_5_ ∈ [−1, 0] represent OutcomeType1, OutcomeType2, OutcomeType3, OutcomeType4, OutcomeType5, respectively, and *a*_1_, *a*_2_, *a*_3_, *a*_4_, *a*_5_ are the coefficient of each outcome type.

In this submodel, we categorize the achievement (perceived outcome) into 5 types: ① OutcomeType1: “Friendly VS. Unfriendly” type, e.g., “Friendly” in the outcome of “Tactile Sensing” and “Gestures Recognition” means that the interpretation of the attitude of an autistic child towards the robot would be friendly, and an extreme friendly outcome, a neutral outcome, and an extreme friendly outcome of this type will be 1, 0, and −1, respectively; ② OutcomeType2: “Positive VS. Negative” type, e.g., “Positive” in the outcome of “Facial Expressions Recognition,” “Natural Language Processing”, and “Contextual Cues” means that, the emotional valence would be positive (e.g, output of a “dislike” in “Natural Language Processing” will be categorized as “Negative”), and an extreme positive outcome, a neutral outcome, and an extreme negative outcome of this type will be 1, 0, and −1 respectively; ③ OutcomeType3: “Valid VS. Invalid” type, e.g., “Valid” in the outcome of “Image Captioning” and “Intention Understanding” means that an autistic child will react positively after the robot verbally described the objects in the image or the robot verbally stated the intention in the interactive scenarios, and an extreme valid outcome, a no feedback outcome, and an extreme invalid outcome of this type will be 1, 0, and −1 respectively; ④ OutcomeType4: “Focused VS. Distracted” type, e.g., “Focused” in the outcome of “Attention Prediction” means that, during the human-robot interaction, the robot can predict that an autistic child has “focused” on one or two objects in the interactive scenario; on the contrary, “Distracted” means the gaze and head direction of the child cannot “fixed on” one or two objects, rather they shifted from one object to another object too often, and “None” means the child cannot “focused” on any object, and an extreme focused outcome, a none outcome, and an extreme distracted outcome of this type will be 1, 0, and −1 respectively; ⑤ OutcomeType5: “Normal VS. Unnormal” type, e.g., “Unnormal” in the outcome of “Action Recognition” and “Noise and Disturbance” means that the robot can detect some abnormal/unhealthy behavior (e.g., “walking on tiptoe” or “having back pain”) of the child or some noise/disturbance in the interactive scenarios, and a normal outcome, and an extreme unnormal outcome of this type will be 0 and −1, respectively.

Note that the probability of simultaneous occurrence of most of or all of these types of outcomes is very low, and usually only a few of them will occur. For each kind of the pattern recognition (i.e., pattern recognition in submodels “local pattern recognition” and “cloud-based pattern recognition”) and the sensing of the environment (i.e., the sensing in submodels “noise and disturbance” and “contextual cues”), as described in the above submodels, the outcome value of which will be mapped into [−1, 1] or [−1, 0] using fuzzy sets depends on which type of outcome is categorized as follows.

Similarly, *PF*_*gc*_, *PF*_*pc*_, PF_*c*_, PF_as_, PF_ao_, PF_ac_, *PF*_*u*_, *PF*_*mv*_ can be defined as follows:

(20) $$\begin{array}{c} {PF}_{gc}=a_{1}\cdot {OT}_{1}+a_{2}\cdot {OT}_{2}+a_{3}\cdot {OT}_{3}+a_{4}\cdot {OT}_{4}+a_{5}\cdot {OT}_{5}\text{,} \end{array}$$

(21) $$\begin{array}{c} {PF}_{pc}=b_{1}\cdot {OT}_{1}+b_{2}\cdot {OT}_{2}+b_{3}\cdot {OT}_{3}+b_{4}\cdot {OT}_{4}+b_{5}\cdot {OT}_{5}\text{,} \end{array}$$

(22) $$\begin{array}{c} {PF}_{c}=c_{1}\cdot {OT}_{1}+c_{2}\cdot {OT}_{2}+c_{3}\cdot {OT}_{3}+c_{4}\cdot {OT}_{4}+c_{5}\cdot {OT}_{5}\text{,} \end{array}$$

(23) $$\begin{array}{c} {PF}_{as}=d_{1}\cdot {OT}_{1}+d_{2}\cdot {OT}_{2}+d_{3}\cdot {OT}_{3}+d_{4}\cdot {OT}_{4}+d_{5}\cdot {OT}_{5}\text{,} \end{array}$$

(24) $$\begin{array}{c} {PF}_{ao}=e_{1}\cdot {OT}_{1}+e_{2}\cdot {OT}_{2}+e_{3}\cdot {OT}_{3}+e_{4}\cdot {OT}_{4}+e_{5}\cdot {OT}_{5}\text{,} \end{array}$$

(25) $$\begin{array}{c} {PF}_{ac}=f_{1}\cdot {OT}_{1}+f_{2}\cdot {OT}_{2}+f_{3}\cdot {OT}_{3}+f_{4}\cdot {OT}_{4}+f_{5}\cdot {OT}_{5}\text{,} \end{array}$$

(26) $$\begin{array}{c} {PF}_{u}=g_{1}\cdot {OT}_{1}+g_{2}\cdot {OT}_{2}+g_{3}\cdot {OT}_{3}+g_{4}\cdot {OT}_{4}+g_{5}\cdot {OT}_{5}\text{,} \end{array}$$

(27) $$\begin{array}{c} {PF}_{mv}=h_{1}\cdot {OT}_{1}+h_{2}\cdot {OT}_{2}+h_{3}\cdot {OT}_{3}+h_{4}\cdot {OT}_{4}+h_{5}\cdot {OT}_{5}\text{.} \end{array}$$

#### 5.2.18. The 18^th^ Submodel “Contextual Understanding of Scenarios and Users”

As illustrated in Figure 6, the outcome of each kind of the pattern recognition and the sensing of environment will be uploaded to the “cloud medical robot platform,” more specifically, to this submodel and the next submodel “cloud-based evaluation system.” As such, in this submodel, our proposed model AppraisalCloudPCT will output the outcome of “contextual understanding of scenarios and users,” which is defined as CUSU = (US, UU), where US represents the understanding of scenarios provided mainly by the output of image captioning and of sensing the environment (i.e., combing scene description with the sensing of noise and disturbance, and of contextual cues), and UU represents the understanding of users provided mainly by the output of other local and cloud-based pattern recognition (i.e., gaze estimation, intention, gestures).

#### 5.2.19. The 19^th^ Submodel “Cloud-Based Evaluation System”

The importance of this submodel is to provide insights into both the cognitive and behavioral status of an autistic child, and of the intention of the child to engage with the robot, to the submodel “cloud-based interaction strategies.”

In this submodel, a personalized machine learning (ML) framework, so-called the personalized perception of affect network (PPA-net) developed by an MIT research group [123], will be adopted in the “cloud-based evaluation system.” As illustrated in Figure 7, in the modified PPA-net, group-level perception of affect network (GPA-net) is trained with the data exacted from the autistic rating scales provided by the doctor or therapist of the child, and the data exacted from the personality profile of the child provided by the parents of the child. Consequently, by using the modified PPA-net, this submodel can automatically provide a continuous and simultaneous estimation of levels of engagement and affective states (i.e., arousal and valence) of an autistic child, to the submodel “cloud-based interaction strategies.”

#### 5.2.20. The 20^th^ Submodel “Information Sharing Between and Learning from Robots”

As mentioned earlier in chapter 4.1.2, one advantage in the research on cloud medical robots is data of interaction between a user and a robot can be stored and evaluated in a cloud for further assessment of the social and communicative characteristics of a user. With the cloud medical robot platform, in this submodel, information (e.g., the personality profile of each autistic child) can be shared between robots with the support of the submodel “cloud-based evaluation system,” and the capability of interpretation of a user and of making decisions can be learned with the support of the submodel “contextual understanding of scenarios and users.”

## 6. Conclusions, Discussion, and Future Work

### 6.1. Conclusions

In this article, we present a novel computational model of emotions so-called AppraisalCloudPCT for socially interactive robots, especially for robots for a special group of people such as autistic children. This model takes into account the social and communicative characteristics of autistic children so that it can fit the need of the autistic children. It mainly results from that our proposed model not only has solid theoretical ground built on a component view of computational models, the appraisal theories on emotions, cloud robotics, and perceptual control theory on emotions but also can be implemented in a social robot developed by us for autistic rehabilitation by adopting mood equation, emotion equation, and personality equation.

Moreover, compared to other significant computational models of emotions for socially interactive robots, our proposed model AppraisalCloudPCT has a number of merits. First, our proposed model can guarantee sufficient rounds of recursive interaction between a robot and an autistic child, so that the interaction will be more effective, efficient, and easier to be satisfied by the child. Second, with our proposed model, a robot can simulate the whole process of human emotion (e.g., generation, regulation, and responding to a stimulus) to a great extent. Third, our proposed model can facilitate sharing information between and learning from various socially interactive robots. Last but not least, our proposed model can be highly computable so that it is suitable to be implemented in various socially interactive robots.

### 6.2. Limitations

Our proposed model AppraisalCloudPCT is designed based on Marsella et al.'s compositional view of model building [5], which lays stress on that emotional models are often composed of individual “submodels” or “smaller components” that can be matched, mixed, or excluded from any given implementation and are often shared. According to Marsella et al. [5], components may be evaluated and subsequently abandoned or improved due to ongoing evaluations before the final version of the model is designed. Although our model is completely designed, there is still room for finding alternative or better mathematical definitions, equations, or algorithms for realizing each individual “submodels.”

### 6.3. Discussion

In this article, we proposed a novel computational model of emotions called AppraisalCloudPCT and elaborated on how to implement it in a socially interactive robot we developed for autistic rehabilitation. However, there are several points that are worthy of being addressed as follows.

First of all, this study is aimed specifically at designing the computational model of emotions for autistic children-robot interaction for three reasons as follows. (1) Although minimal progress has been made in advancing the clinical use of robotics in ASD interventions in clinical settings [13], applying robots for autism interventions still achieved a number of targets [11], and 24 of 74 ASD objectives in the “eight domains” as mentioned in Section 1 can potentially be applied to. (2) Modeling of emotions is of critical importance for robots when interacting socially with humans [8]. This is so because the robot's emotional responses are determined by the robot's computational model of emotion, in the light of its own internal cognitive-affective state and its interactions with the external environment [7]. (3) There are four world-leading research groups with pioneering work in promoting social robots as useful tools in autism therapy, but none of them have designed or applied computational models of emotions for the social robots used in their autism therapy studies.

Second, in Section 4.2, we chose the five crucial properties of a computational emotion model as listed in [103], alongside with one more property, i.e., combining with cloud robotics, to be the six criteria for comparison. We believe that “combining with cloud robotics” can be a crucial property of a computational emotion model, and it can be a fair criterion for comparison of computational emotion models to make robots smarter and better satisfied by the users, as well as to promote sales in the service robots market for three reasons as follows. (1) As mentioned before, a computational model of emotions should endow a robot with a more powerful capability of making decisions faster, more appropriate, and more efficient, given that more and more socially interactive robots will be exposed to various users with different backgrounds and be connected to substantial Internet of Things (IoT) such as medical IoT with massive medical data. As “combining with cloud robotics” can facilitate sharing information between and learning from socially interactive robots, we believe that this property will be crucial in building the computational models. (2) Given that other crucial properties such as (iv) data-driven mapping and (v) ethical reasoning are heavily data-driven and in great demand of computing power, “combining with cloud robotics” could be an efficient if not the best way to guarantee that data consisting of interaction between a user and a robot can be stored and evaluated in a cloud for further assessment of the social and communicative characteristics of a user. (3) On one hand, more and more socially interactive robots are implemented artificial intelligence (AI) algorithms or deep learning (DL) (e.g., the modified PPA-net implemented in our own robot)/reinforcement learning (RL)/deep reinforcement learning (DRL) frameworks to make them smarter and better received by the users; on the other hand, deploying them in the main controller of a robot rather than in a cloud will increase the hardware cost due to increased computational load. Since the parents of autistic children usually suffer from heavy burden not only mentally but financially, “combining with cloud robotics” would be necessary for promoting robots with acceptable prices in the service robots market to those parents.

Third, our proposed model AppraisalCloudPCT could be implemented in a socially interactive robot that we developed for autistic rehabilitation. Such a model could also be adapted to service people with different special needs, e.g., dementia elders. This results from that our proposed computational model of emotions takes into account the social and communicative characteristics of a special group of users such as autistic children or dementia elders, through its submodel so-called cloud-based interaction strategies, which is supported by two submodels (i.e., “contextual understanding of scenarios and users” and “cloud-based evaluation system”) in a cloud medical robot platform, as illustrated in Figure 1. As mentioned before, a cloud-based evaluation system enables the data of interaction between a user and a robot to be stored and evaluated in a cloud for further assessment of the social and communicative characteristics of a user. Furthermore, the submodel “contextual understanding of scenarios and users” relies on another two submodels “local pattern recognition” and “cloud-based pattern recognition,” which can provide the interpretation of a user's intention, attention, emotional state, and behavior. Therefore, the proposed computational model of emotions is suitable for socially interactive robots, particularly robots for a special group of users such as autistic children or dementia elders, which promote the universality of our model to some extent. Moreover, our proposed computational model also meets the criteria of “domain-independent,” i.e., processing and exhibiting emotional responses in various situations as well as in certain kinds of interaction domain, since it can coordinate a robot implemented with our model to respond to the surrounding emotional contexts appropriately.

For our proposed model to be adapted to socially interactive robots servicing dementia elders, a few steps would be necessary as follows. (1) As illustrated in Figure 7, a group-level perception of affect network (GPA-net) in the modified PPA-net will be trained with the data exacted from the dementia rating scales such as mini-mental state examination (MMSE) [127] provided by the doctor or therapist of the dementia elder, and the data exacted from the personality profile of the dementia elder provided by the offspring or close friends of the dementia elder. Consequently, by using the modified PPA-net, this submodel can automatically provide simultaneous and continuous estimation of the different levels of affective states (i.e., valence and arousal) and engagement of a dementia elder, to the submodel “cloud-based interaction strategies.” (2) With the support from the two submodels “cloud-based evaluation system” and “contextual understanding of scenarios and users” which can provide the specific estimation of valence, arousal, and engagement levels of the dementia elder, and the contextual understanding of the interactive scenario and the dementia elder, respectively, the robot will be able to alter its mood and personality to match with the status of the dementia elder and the interactive context using the three interaction strategies in the submodel “cloud-based interaction strategies,” for a better round of interaction. (3) Our proposed model is designed in the first place to enable many rounds of recursive interaction between a robot and a user. Based on the feedback (e.g., the interpretation of a dementia elder's intention, attention, emotional state, and behavior) provided by the dementia elder during/after the human-robot interaction, as summarized by the submodel “achievement (perceived outcome)”, the three interaction strategies in the submodel “cloud-based interaction strategies” can be modified accordingly. As such, the interaction will be more effective, efficient, and easier to satisfy the needs of dementia elder after many rounds of recursive interaction.

### 6.4. Future Work

Future studies should examine how our model performs in various robots and in more interactive scenarios.

## Figures and Tables

**Table 1:** Comparison of six computational models of emotions for socially interactive robots.

Models	Criteria					
	Domain-independent	Models mood	Models personality	Data-driven mapping	Ethical reasoning	Combines with cloud robotics
Kismet	√	?	×	√	√	×
WE-4RII	√	√	√	×	?	×
PRESENCE	√	×	×	√	√	×
iGrace	√	√	√	√	√	×
xEmotion	√	√	√	√	?	×
Our model	√	√	√	√	√	√

*Note*. (1) A model that satisfies the given property is marked with a tick mark (√); a model that does not satisfy the given property is marked with a cross mark (×), and when we were unable to retrieve enough information to determine whether a specific property was met, we use a question mark (?). (2) According to Ojha et al. [103], “domain-independent” means processing and exhibiting emotional responses in various situations but not only in certain kinds of interaction domain; “models mood” means integrating the notion of mood with emotions; “models personality” means integrating the notion of personality; “data-driven mapping” is defined as a data-driven mapping of the appraisal variables into emotion intensities according to the learned relationship between emotions and appraisal variables; as for “ethical reasoning,” it is defined to be an emotion regulation mechanism implemented based on ethical reasoning for the emotional and behavioral responses of social robots to be more “acceptable” in the human community.

**Table 2:** A summary of the six key capabilities of our developed robot Dabao in interactive scenarios with Chinese autistic children.

Interactive scenarios	Capabilities		
	Perception (i.e., to perceive an autistic child via cameras, tactile sensor arrays, microphones, etc.)	Cognition (i.e., to infer an autistic child's mood and cognitive states, and behavior, etc.)	Action (i.e., to express itself to an autistic child through facial expressions, gestures, speech, etc.)
Recognizing the gestures of an autistic child and imitating the user	The robot can recognize five hand gestures (e.g., OK, punch) and ten body gestures (e.g., kick, wave)	There is a mapping between the skeleton feature key points of an autistic child and the output gestures of the robot	The robot can either imitate the gestures of the autistic child or feedback to the child with one of the seven emotional body gestures accordingly
Chatting with an autistic child [107]	Via automatic speech recognition (ASR)	Via natural language processing (NLP)	Via text-to-speech (TTS)
Tactile sensing of an autistic child and responding to the child accordingly	The robot can sense four types of primary tactile characteristics (i.e., area, location time, and direction) of an autistic child via 21 tactile sensor arrays by our own design [106]	Ten machine learning algorithms including SVM and KNN are selected using K-fold cross-validation to classify the 6 touch behaviors (e.g. finger sliding) of the child	There is a mapping between an event-triggered tactile perception and the output (facial expressions, gestures, speech, etc.) of the robot
Recognizing the facial expressions of an autistic child and responding to the child accordingly	Via our lightweight CNN architecture DeepLook [105] or Concat-Xception [111] that runs on Jecton TX2, our robot can recognize the 6 basic facial expressions (e.g., anger) of a child at an average rate over 70%	There is a mapping between the facial expressions of a child perceived by the robot and the output facial expressions of the robot that takes into account the child's ability to perceive	An appropriated facial expression out of 20 choices (shyness, thinking (turning eyes), etc.) will be displayed to the child with body gestures and speech output sometimes
Attention-based image captioning with gaze-following [110]	A robot takes a video as input, then builds a 3D dense map based on SLAM, and estimates the gaze simultaneously	Attention prediction is done by attention heat map adding occlusion and salience detection	The robot verbally describes the region focused on by the child based on attention heat maps
Intention understanding based on visual-NLP semantics and responding to the child accordingly [109]	The robot extracts information from one of the three types (i.e., vague, feeling, and clear) of instructions from a child and identifies the objects in front of the robot	The robot infers the intention of the child by a similarity computation algorithm and then transforms it into a structured robot control language	Turning itself to face the child, the robot points out a target object with a verbal description of the intention of the child

## Data Availability

All data included in this study are available from the corresponding author upon request.
